# Consecutive Bilateral Iliac Stress Fracture in an Adult Male Runner

**DOI:** 10.7759/cureus.59013

**Published:** 2024-04-25

**Authors:** Amr Chaabeni, Amine Kalai, Imen Dghim, Mezri Maatouk, Anis Jellad

**Affiliations:** 1 Physical Medicine and Rehabilitation, Faculty of Medicine, University of Monastir, Monastir, TUN; 2 Radiology, Faculty of Medicine, University of Monastir, Monastir, TUN

**Keywords:** magnetic resonance imaging, sport, runner, athlete, iliac bone, stress fracture

## Abstract

Iliac stress fractures (ISF) are uncommon in sports, particularly among runners, and are attributed to repetitive loading and other predisposing factors. ISF poses diagnostic challenges due to nonspecific symptoms and the limited sensitivity of conventional imaging procedures. The reported case is about a 51-year-old male marathon runner with consecutive bilateral ISF. Initial symptoms included mechanical pain in the right buttock, leading to a diagnosis confirmed via pelvic MRI. Conservative management with eight weeks rest from sport activity was indicated with symptom resolution and return to sport. However, 20 days after returning to sport, the patient developed left-sided symptoms post-resumption of running, with MRI confirming a new ISF. An additional eight weeks of rest was prescribed, allowing the patient to resume sport at preinjury levels. ISF should be considered in runners presenting with gluteal pain, emphasizing the importance of early diagnosis. MRI emerges as a valuable tool for accurate diagnosis, guiding appropriate management strategies. Conservative management focusing on rest is paramount for favorable outcomes and optimizing runners' health and performance.

## Introduction

Stress fractures are notable among elite athletes and military recruits, with a prevalence of 21%, and often affect the lower limb bones [1]. The specific bones involved often correlate with the type of sporting activity; for instance, throwing sports may involve the humerus, whereas runners commonly experience stress fractures in the lower extremities. The iliac bone is a relatively uncommon site for stress fractures. The pathophysiology of iliac stress fractures (ISF) in sports involves repeated loading of bone, resulting in loss of normal bone metabolism and failure of remodeling [1]. Repeated mechanical loading can lead to a disconnection between osteoblast-mediated bone formation and osteoclast-mediated bone resorption. In general, factors such as repetitive impact through sports or military training, as well as metabolic abnormalities, nutrient deficiencies, and genetic predisposition, contribute to the risk of developing a stress fracture [2,3]. Hence, runners are predisposed to a heightened risk of stress fractures [4]. Contributing factors include prior injury, participation in marathon training, choice of footwear, and running kinematics [5]. The occurrence of stress fractures in athletes is also influenced by specific factors such as muscle attachments in the surrounding area, muscle fatigue, which can result in the transmission of excessive forces to the underlying bone, and the nature of athletic activity. The diagnosis of ISF can be challenging because it may not be detected during standard diagnostic evaluations and imaging procedures [6]. Few studies have reported the occurrence of iliac bone stress fractures in athletes, particularly marathon runners [7-10].

Here, we present the case of an adult male marathon runner with consecutive bilateral ISF without a previous injury or other medical history.

## Case presentation

A 51-year-old veterinarian, with eight years of regular running practice comprising seven hours (80 km) per week, presented with a two week history of mechanical pain localized to the right buttock region. The patient's medical history was unremarkable, except for a prior resolved episode of nonspecific low back pain.

**Figure 1:**
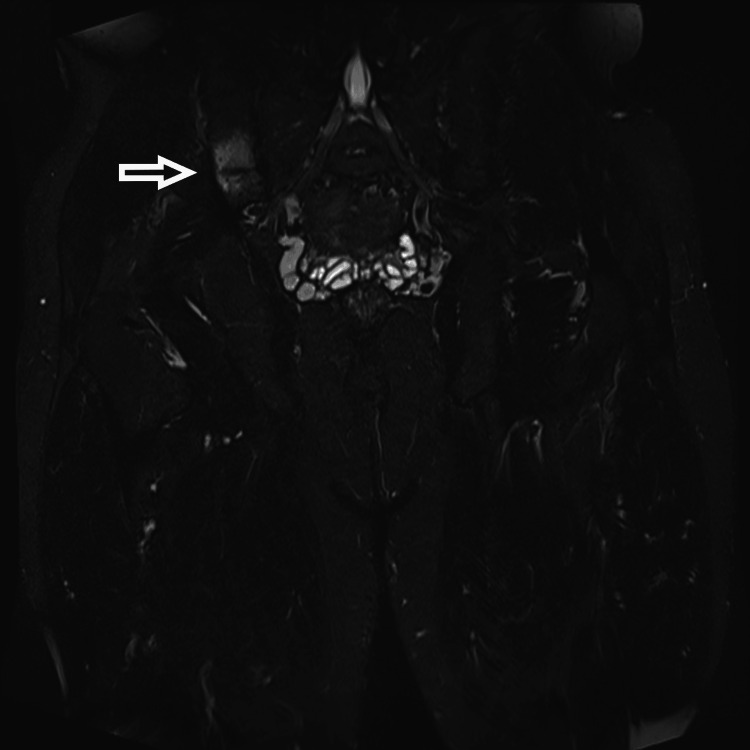
Initial MRI with coronal view STIR showed a partial fracture of the right ilium with bone marrow oedema (arrow) STIR - Short Tau Inversion Recovery

On anamnesis, the patient recognized the onset of pain after 15 min of running or hiking, with limping forcing him to stop the effort. On physical examination, pain in the right gluteal area was triggered on palpation and the ipsilateral single-leg hop test. No other abnormalities were found, notably in neurological, lumbar spine, and hip examinations. Laboratory tests revealed no biological inflammatory or bone metabolic abnormalities. Pelvic magnetic resonance imaging (MRI) revealed right iliac bone marrow oedema next to the sacroiliac joint (Figure 1).

MRI allowed us to rule out pelvic soft tissue, lumbar spine, and femoral head disease. The diagnosis of a stress fracture of the right iliac bone was confirmed.

An eight week rest from sports activity was indicated, and the pain was treated with painkillers on demand. The evolution of symptoms was favorable, and the patient resumed sports activity at the same level at the end of the prescribed rest period. 

**Figure 2:**
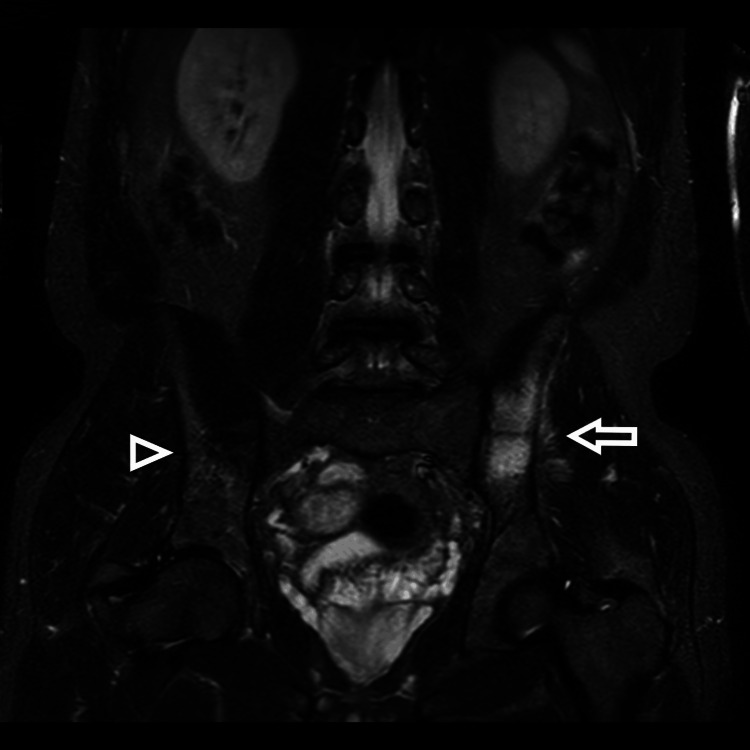
Second MRI coronal view with STIR TSE, revealed a second symmetric contralateral ilium fracture (arrow) with partial regression of oedema around the initial right ilium fracture (arrowhead). STIR-Short Tau Inversion Recovery, TSE-Turbo Spin Echo.

The patient visited us 20 days after resuming sports activity, with pain in the left gluteal region. A second pelvic MRI revealed a stress fracture on the iliac side of the left sacroiliac joint with healing signs of the previous right iliac fracture (Figure 2).

An additional eight week rest from sports activity was prescribed until the pain subsided; then, the patient resumed running progressively over three weeks, with a gradual increase to the preinjury level. The patient was satisfied with the results and had recently participated in a desert trek (100 km) without discomfort.

## Discussion

Our case stands out for, the absence of stress fracture risk factors (apart from running activity), the occurrence of subsequent bilateral ISF, the favorable outcome marked by symptom improvement after rest, gradual return to running, and long-term satisfaction. Stress fractures in the pelvis, including those in the iliac bone, comprise only 1-7% of all stress fractures [11]. Stress fractures in the iliac bone, as diagnosed in our case, are particularly rare, with an estimated prevalence of approximately 4% of all pelvic stress fractures [12]. These fractures warrant consideration, particularly among long-distance runners who are predisposed to such injuries, as exemplified in our case [11]. It has been reported that run athletes, as in our case, can experience ISF without any underlying disease [10]. During weight-bearing sports activities, such as running and jumping, ISF may occur due to the significant shear forces exerted on the pelvic region. It has been reported that in a mechanical sense, iliac wing failure may occur gradually owing to the repetitive stresses imposed by the upward traction force of the abdominal muscles and the counteracting force of the abductors, both of which are inserted on the iliac crest [8].

ISF can easily be missed during routine workups and imaging [13]. Typically, they occur in the superomedial region of the iliac bone and present with vague symptoms, often resulting in a benign physical examination [14].

Early detection and conservative treatment are important for symptom resolution [11]. Nonetheless, diagnosing these fractures can be challenging owing to the nonspecific nature of symptoms and the absence of notable findings upon physical examination [6].

In our case, the presented symptom was mechanical pain in the buttock region without any significant medical history. Physical examination yielded limited results, demonstrating nonspecific pain upon palpation and during the single-leg hop test. Therefore, symptoms and physical examination findings alone lack specificity and adequacy for the diagnosis of ISF. Diagnosis commonly relies on radiographic examination, particularly MRI. Conventional radiographs demonstrate limited sensitivity in detecting early stress fractures, particularly those involving the iliac bone [15]. In contrast, MRI is highly useful in the diagnosis and management of stress fractures. It allows for the assessment of the severity and location of the fracture, as well as differentiation from other conditions. With its high sensitivity and specificity, MRI stands out as the preferred imaging modality for detecting stress fractures [8, 16]. This was exemplified in our patient's case, where MRI successfully identified consecutive bilateral ISF and also revealed signs of healing in the previously sustained fracture.

Conservative management, primarily emphasizing rest from athletic endeavors, is the cornerstone of treatment for ISF, typically resulting in favorable outcomes. To ensure successful management, physical therapy primarily involves core stability and hip girdle strengthening exercises, monitoring the return to sports activities, and incorporating non-impact aerobic exercises, followed by a gradual progression to running [9]. This may be because runners usually return to sports within a period of four weeks [17]. However, surgery may be necessary in cases of non-union or delayed union [18].

## Conclusions

ISF should be considered in runners who present with mechanical pain in the gluteal region. Early-stage diagnosis is crucial, and MRI should be promptly employed to confirm the condition, facilitating the initiation of appropriate treatment. Primary treatment focuses on resting from athletic activities to promote bone healing.
